# Shear characteristics and stress-strain mathematical model of waste polyester textile reinforced clay

**DOI:** 10.1038/s41598-020-62168-8

**Published:** 2020-03-23

**Authors:** Xiangfeng Lv, Hongyuan Zhou

**Affiliations:** 1 Beijing Key Laboratory of Urban Underground Space Engineering, School of Civil and Resource Engineering, University of Science & Technology Beijing, Beijing, 100083 China; 2 Key Laboratory of Ministry of Education on Safe Mining of Deep Metal Mines, College of Resources and Civil Engineering, Northeastern University, Shenyang, 110819 China

**Keywords:** Civil engineering, Mechanical properties

## Abstract

Clay reinforcement through appropriate applications of waste fiber or waste fiber fabric can generate huge economic and environmental benefits. In this study, clay was reinforced using waste polyester fiber filaments and waste polyester fabric blocks, respectively. Triaxial tests (*σ*_1_ > *σ*_2_ = *σ*_3_) were carried out to examine the influence of reinforcement method and the contents (0.0%, 0.5%, 1.0%, 1.5%) on the shear behavior of clay. After reinforcement, the deformation resistance and shear strength of the clay was improved. The optimal contents of fiber filament and fabric block were both 1.0%; as the fiber filament or fabric block content increased from 0.5% to 1.5%, the stiffness of the reinforced clay decreased, while the energy absorption capacity and the cohesive strength first increased and then decreased. Under the optimal content condition, the fiber filament showed better reinforcement than the fabric block. Under the train hardening condition, a hyperbolic model can be used to quantitatively describe the stress-strain relationship of the reinforced clay, and the model parameters can also reflect the strain hardening degree.

## Introduction

Soft clay is widely available in the eastern coastal areas of China. Due to its low bearing capacity and large deformability, it often leads to large settlement of overlying buildings, which seriously threatens the stability and safety of engineering structures and greatly restricts economic and infrastructure development in the region^1,2^. Therefore, it is of great significance to take appropriate reinforcement measures to improve the engineering mechanical properties of soft clay to make it meet the requirements for such applications.

At present, there are many methods for soil improvement, including replacement, strong tamping, grouting, and reinforcement using fiber materials^3^. Among these, fiber reinforcement can significantly improve the peak compressive strength and shear strength of clay^4,5^, effectively inhibit clay cracking and deformation^6,7^, increase the ductility of clay, and reduce the post-peak strength loss^8^. It has the advantages of simple on-site operation, low cost, and wide application range, and has become the most commonly-used method in this type of engineering^9^. Fiber reinforcement refers to a soil mass improvement technique in which dispersed fiber filaments are uniformly incorporated into the soil mass to improve its engineering mechanical properties^10^. Currently, the fibers used in engineering projects mainly include natural fibers, synthetic fibers, and recycled fibers from waste materials. Among these, natural fibers are obtained from the local ecological environment where the engineering project is located. These include bamboo fiber, hemp fiber, plant roots, and animal or human hair, among others^11^. Synthetic fibers mainly include polypropylene fiber, glass fiber, carbon fiber, and basalt fiber^12^. Recycled fibers include polyester fiber, nylon fiber, cotton fiber, and rubber fiber^8^. In recent years, low cost and green reinforcement of clay has become a hot topic in geotechnical engineering.

Mirzababaei *et al*. 2013 found that the fibers prepared from the waste carpet could significantly improve the unconfined compressive strength, reduce the rate of post-peak strength decay, and increase the ductility of the clay^8^. Cao *et al*. 2014 used cotton fiber fabric blocks (12 mm in length, 5 mm in width) and polyester fiber fabric blocks (12 mm in length, 5 mm in width) prepared from waste clothes to modify clay^13^. By implementing the cutting-ring based penetration test, California bearing ratio (CBR) test, and direct shear test, both the above two types of fabric blocks prepared from the waste clothes could significantly improve the shear strength and bearing capacity of the clay. Estabragh *et al*. 2012 used nylon fibers to reinforce cement-solidified clay and studied the effects of fiber content, cement content, and curing age on the unconfined compressive strength. He pointed out that nylon fiber could increase the unconfined compressive strength and peak strain of the cement-solidified clay, which resulted in the brittle-ductile failure transition of the cement-solidified clay^14^. Consoli *et al*. 2002 used plastic chips prepared from discarded plastic bottles to reinforce cement-solidified sand. The effects of plastic chip content, size, and cement content on the deformation and strength characteristics of cement-solidified sand were studied using triaxial shear tests^15^. Chen *et al*. 2015 used fiber fabric blocks prepared from waste polypropylene fiber bags to reinforce cement-solidified sand, and determined the effects of fiber content, fiber length, and curing age on unconfined compressive strength^5^. Foose *et al*. 1996 pointed out that the tire shreds content, initial sand density, and normal stress were the key factors affecting the shear strength^16^. Meanwhile, Hataf *et al*. 2006 noted that the bearing capacity reached its peak under the optimal content and optimal length based on CBR test results, which showed the best reinforcement effect^17^.

According to the statistics and analysis of prior studies, both waste fiber and waste fiber fabric possess high tensile strength and good resistance to deformation and can be used for harmless and green reinforcement of soil^18,19^. According to limited statistics of Liu *et al*. 2017, the cumulative production of waste fiber fabrics in China reached 140 million tons with a <10% recycling rate between 2011 and 2015. The annual fiber fabric consumption growth rate is >12%^20^. The appropriate disposal and recycling of waste fiber fabrics have long been a difficult problem in the field of environmental protection. Therefore, the application of waste fiber or waste textile fiber fabrics in soil reinforcement will bring great environmental and economic benefits. However, there are few reports on the reinforcement of soil by using waste fiber or waste fiber fabric and a comparative study on their reinforcement effects is still missing.

This study aims to promote the application of waste polyester fiber or waste polyester fabric blocks in clay reinforcement. The clay was reinforced with either waste polyester fiber filament or waste polyester fabric blocks. Under the conditions of optimum moisture content and maximum dry density, the influences of the reinforced method, waste polyester fiber filament content (0.0%, 0.5%, 1.0%, 1.5%), and waste polyester fabric block content (0.0%, 0.5%, 1.0%, 1.5%) on the failure modes, stiffness, energy absorption capacities, and shear strength were studied using triaxial shear tests. A mathematical model of the stress-strain relationship of reinforced clay was established based on these test results.

## Materials and methods

### Basic physical and chemical properties of clay

#### Physical properties

**Figure 1:**
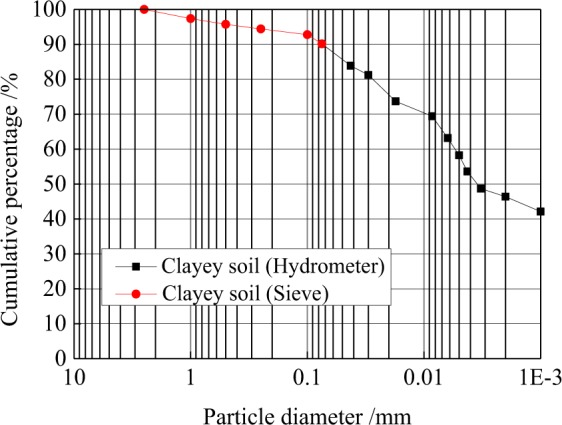
Particle size distribution of the clay.

The clay used in this study was taken from the construction site of an underground integrated pipe gallery in Beijing, China. The physical properties of the clay are reported in Table 1. The specific gravity, Gs, of the clay was determined using helium gas pycnometry (Pycnomatic ATC), according to^21^. The particle size distribution of the soil is presented in Fig. 1. Various percentage fractions such as gravel (N4.75–b19 mm), sand (N0.075–b2 mm), silt (N0.002–b0.075 mm) and clay (b0.002 mm) were determined according to ASTM D 422-63 (1994). The Atterberg limits were determined according to^22^ and^23^ and the soil was classified as clay of low plasticity, CL, according to the Unified Soil Classification System (USCS).

**Table 1:** Basic physical properties of clay.

Categories	CL
Specific gravity	2.65
Gravel content (%)	0.0
Sand content (%)	9.8
Slit content (%)	31.9
Clay content (%)	58.2
Liquid limit (%)	26.5
Plastic limit (%)	15.7
Plasticity index (%)	11.8
Maximum dry density (kN/m^3^)	16.7
Optimum moisture content (%)	15.5

#### Mineral and chemical compositions

The mineral and chemical compositions of the clay were determined by X-ray diffraction (XRD) and X-ray fluorescence spectrometry. The results are shown in Table 2.

**Table 2:** Statistics on mineral composition and chemical composition of clay.

	Quartz	Sodium feldspar	Chlorite	Illite	Kaolinite	Calcite
Mineral composition (%)	72.28	14.98	3.70	2.03	1.77	5.24
Chemical composition (%)	SiO_2_	Al_2_O_3_	CaO	MgO	—	—
	52.2	15.2	6.3	8.3	—	—

### Basic physical and mechanical properties of waste textile fibers

**Figure 2:**
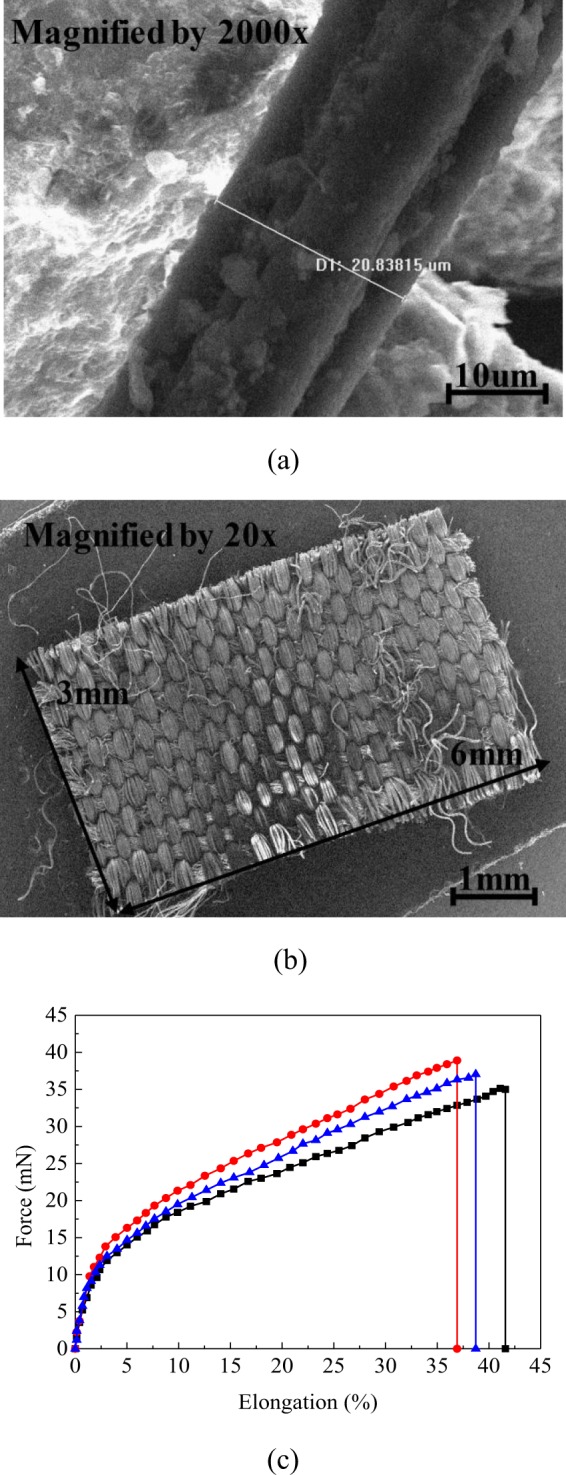
(**a**) SEM image of the polyester fiber used in the study. (**b**) SEM image of the fabric block used in the study. (**c**) Typical force-strain curve of the polyester fiber used in the study.

The waste polyester fiber filaments were obtained by cutting the fiber filament using a fiber cutting machine, as shown in Fig. 2(a). The waste polyester fabric blocks were obtained by cutting the waste polyester fabric also using the same machine, as shown in Fig. 2(b).

The mechanical properties of the polyester fiber were tested using a fiber tensile test apparatus (Textechno Fafegraph ME, Germany) according to^24^ ASTM D 1577-07 and^25^ ASTM D 3822. In order to ensure the accuracy of the test results, three consecutive tensile tests were carried out, and the tensile force-strain curves obtained are shown in Fig. 2(c). The linear density of the polyester fiber was determined by measuring the mass of a 1000 m fiber filament. The mechanical strength of the fiber was further determined in terms of tenacity, which is defined as the breaking force divided by the linear density of the fiber. The physical and mechanical parameters of the fiber are shown in Table 3.

**Table 3:** Physical and mechanical properties of fiber filament.

Specific gravity	Effective diameter (μm)	Linear density (tex)	Breaking force (mN)	Tenacity (mN/tex)	Breakage elongation (%)
1.24	20.83815	0.322	111.07	344.9	38.91

### Sample preparation and test methods

#### Sample preparation

Mirzababaei *et al*. 2013 mixed clay and fibers in three different ways: mixing pre-wetted fibers with dry clay powder, mixing dry fibers with wet clay, and mixing dry clay with dry fibers first then adding water^8^. Based on the test results, they pointed out that the last mixing method could obtain a uniform mixture of fiber and clay. This is because after the fibers are mixed with the clay particles, the latter will form a coating on the surface of the blocks or the fibers, thereby preventing the blocks or the bundles of fibers from stacking together.

The triaxial test samples were 38.1 mm in diameter with a height of 80 mm. The moisture content was the optimum moisture content, and the dry density was the maximum dry density. The fiber filament length was 6 mm, and its contents were 0.5%, 1.0%, and 1.5%. The fabric block size was 6 mm × 3 mm, and its contents were 0.5%, 1.0%, and 1.5%. Before mixing, the methods proposed by Chen *et al*. 2015 and Hamidi *et al*. 2013 were used to determine the mass of clay, fiber, and water^5,26^. The fiber content was defined as the ratio between fiber mass and dry clay mass. When mixing the samples, a certain amount of fiber filaments or fabric blocks were added to the clay over five instances. Each time, the mixture was stirred for 2 min with a stirrer. It should be pointed out that the UJZ-15 mixer produced by China Yongtest Instrument Company was used in the research. The diameter of the stirring blade is 0.23 m, and the rotation speed is 80 ± 4 r/min. The diameter of the stirring cylinder is 0.38 m and the height is 0.29 m. After that, water was added to the mixture of clay and waste polyester fiber or waste polyester fabric and then stirred for 3 min. The prepared mixture was kept in a sealed container for 3 to 4 days to ensure a uniform moisture distribution. It should be noted that the light compaction test was used to determine the optimum moisture content and the maximum dry density^27^. In addition, the moisture of the samples was tested by the drying method.

**Figure 3:**
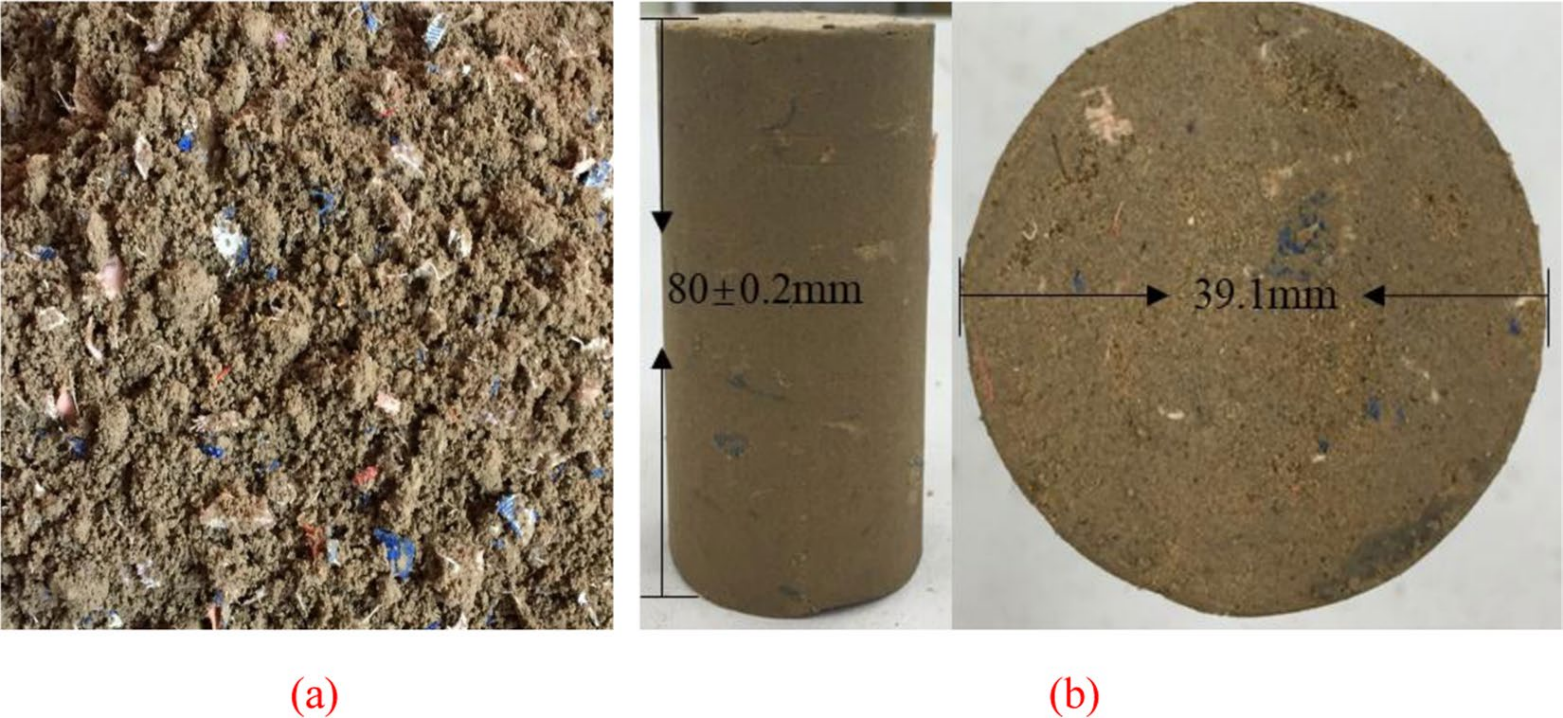
Mixture and test sample (**a**) clay mixed with waste polyester textile (**b**) test sample.

Before preparing the triaxial sample, the moisture content in the mixture was re-measured. The lost moisture was compensated for by steam humidification until the predetermined moisture content was reached. In order to control the dry density of the sample during sample preparation, the mixed reinforced clay was filled into a metal mold (the volume is constant) having a diameter of 39.1 mm and a height of 80 mm in eight phases^28^. After each filling, the clay was hammered 25 to 30 times with a metal hammer. The final sample height was 80 mm ± 0.2 mm. Figure 3 shows the mixture and typical sample.

#### Test methods

Triaxial tests were carried out using a TSZ-1 full automatic strain controlled triaxial test apparatus produced by Nanjing Soil Instrument. The confining pressures were 100 kPa, 300 kPa, and 500 kPa. The shear rate was 0.8 mm/min. The test ended when the strain reached 16%. After the test, the shear strength was determined based on the stress-strain curve characteristics. If a peak occurred on the stress-strain curve before the strain reached 15%, then the stress value corresponding to the peak was considered as the shear strength. Otherwise, the stress value at 15% strain was considered as the shear strength. The triaxial test variables are shown in Table 4.

**Table 4:** Triaxial test variables.

Dry density/(kN/m^3^)	Moisture content/%	Fiber type/%	Size	Content/%	Confining pressure/kPa
16.7	15.5	Filament	Length 6 mm, Effective diameter 20.83815 μm	0.0/0.5/1.0/1.5	100/300/500
		Block	Length 6 mm, Width 3 mm	0.0/0.5/1.0/1.5	100/300/500

## Results and discussion

### Stress-strain curve characteristics

**Figure 4:**
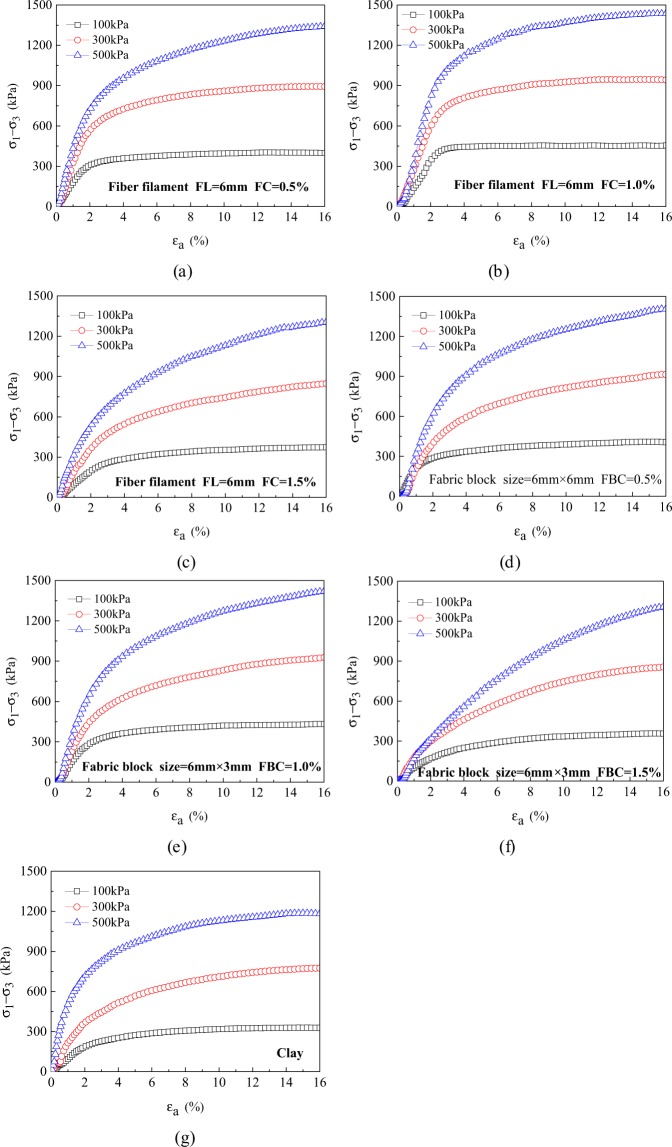
Stress-strain curves of clay and two types of reinforced clays. (**a**) 0.5% of fiber filaments (**b**) 1.0% of fiber filaments (**c**) 1.5% of fiber filaments (**d**) 0.5% of fabric blocks (**e**) 1.0% of fabric blocks (**f**) 1.5% of fabric blocks (**g**) Clay without additives. FC: fiber filaments content FBC: fabric blocks contents.

The stress-strain curves of clay, waste polyester fiber filament reinforced clay, and waste polyester fabric block reinforced clay are shown in Fig. 4(a–g). All three types of clays exhibit a strain-hardening type of response during the triaxial test, such that the stress increased or tended to stabilize with increasing strain. Compared with clay, the two types of reinforced clays showed a more obvious strain-hardening characteristic, i.e., the stress increases were larger at the same strain rate. The above results showed that both the fiber filament and the fabric block could change the load-bearing behavior of the clay under external load and optimize the load-bearing structure of clay. The stress-strain relationships of all three types of clays were also closely related to both the confining pressure and the fiber content; the larger the confining pressure and the higher the fiber content, the more obvious the strain-hardening characteristics.

### Variations of the stiffness and energy absorption capacity of reinforced clay

In order to quantitatively evaluate the influence of fiber filaments and fabric blocks on the stress-strain relationship of clay, the stress-strain curve was used to calculate the stiffness and energy absorption capacities of clay, waste polyester fiber filament reinforced clay, and waste polyester fabric block reinforced clay. The energy absorption capacity can be determined by calculating the area below the stress-strain curve, and reflects the deformation resistance of a sample from the beginning of external loading to the breakage of the sample^26^. In this study, Matlab 2017 was used to calculate the energy absorption capacity. The larger the energy absorption capacity, the better its resistance to deformation. The stiffness is defined as the ratio of the peak stress to its corresponding strain^26^. The smaller the stiffness, the larger the cumulative plastic deformation, i.e., the higher the likelihood of plastic damage.

**Figure 5:**
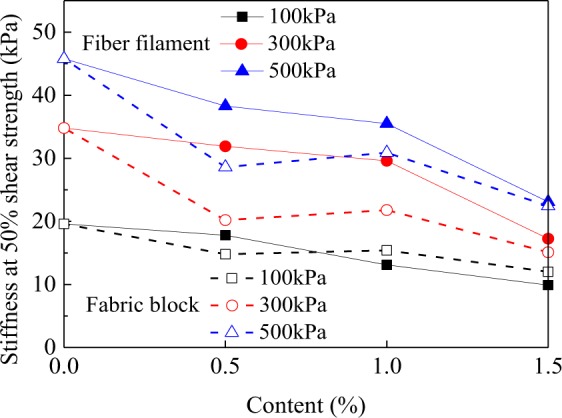
Variations of the stiffnesses of two types of reinforced clays.

Figure 5 shows that for a fixed confining pressure, the larger the fiber filament or fabric block contents, the smaller the stiffness of reinforced clay, and the higher the possibility of plastic damage. When the contents of fiber filaments or fabric blocks were fixed, the stiffness increased with increasing confining pressure, which is consistent with the results of^12^ and^29^. The effect of the reinforcement method on the changes in stiffness can also be obtained from Fig. 4. When the confining pressure is 100kPa, there is no clear relationship between the stiffness of the waste polyester fiber monofilament reinforced clay and that of the waste polyester fiber fabric block reinforced clay. When the confining pressures were 300 kPa and 500 kPa, the stiffness coefficient of the fabric block reinforced clay is significantly larger than that of the fiber filament reinforced clay. This indicated that the cumulative plastic deformation of reinforced clays had a “confining pressure effect,” such that at high confining pressures, the cumulative plastic deformation of fiber filament reinforced clay was larger than that of fabric block reinforced clay.

**Figure 6:**
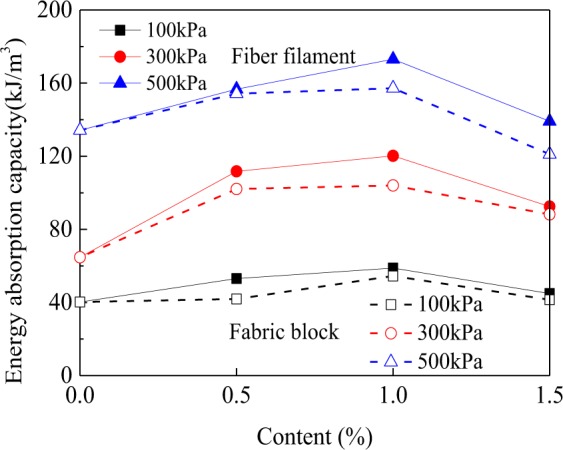
Variations of the energy absorption capacity of two types of reinforced clays.

Figure 6 shows that after the clay was reinforced, the energy absorption capacity significantly increased. When the confining pressure was 500 kPa and the fiber content was 1.0%, the energy absorption capacities of both waste polyester fiber filament reinforced clay and waste polyester fabric block reinforced clay reached a peak value of 173.1 kJ/m^3^ and 157.2 kJ/m^3^, respectively. Compared with the energy absorption capacity of clay (120.2 kJ/m^3^), the energy absorption capacities of the above two types of reinforced clays increased by 44.0% and 30.8%, respectively. When the contents of the fiber filament and fabric block were fixed, the energy absorption capacity increased with increasing confining pressure. The effect of the reinforcement method on the changes in energy absorption capacity can also be obtained from Fig. 6. When the confining pressure, fiber filament content, and fabric block content were fixed, the energy absorption capacity of fiber filament reinforced clay was larger than that of fabric block reinforced clay. This indicated that the overall deformation resistance of the former reinforced clay was better than the latter.

### Failure envelope and shear strength parameter variations of reinforced clay

**Figure 7:**
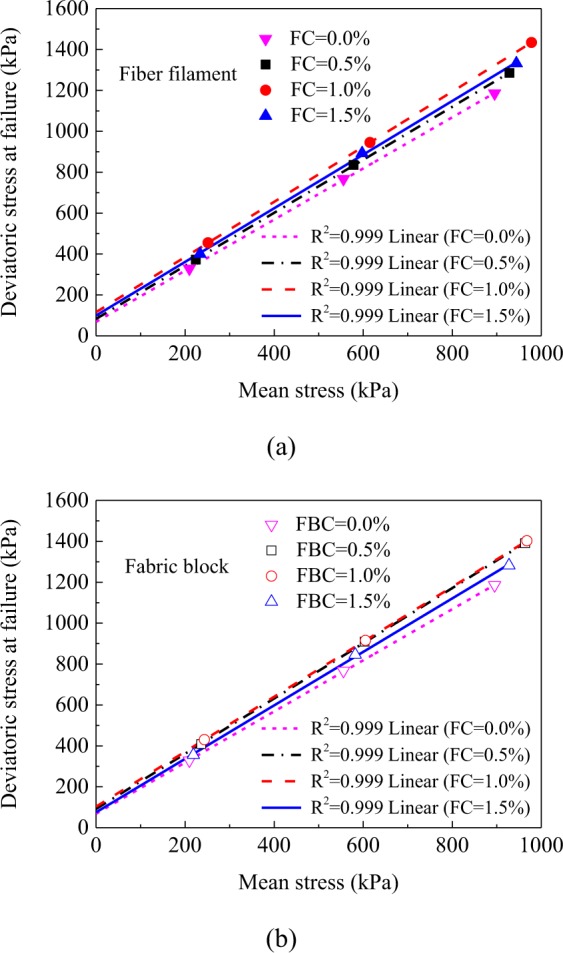
The failure envelopes of two types of reinforced clays. (**a**) Waste polyester fiber filament reinforced clay; (**b**) waste polyester fabric block reinforced clay.

**Figure 8:**
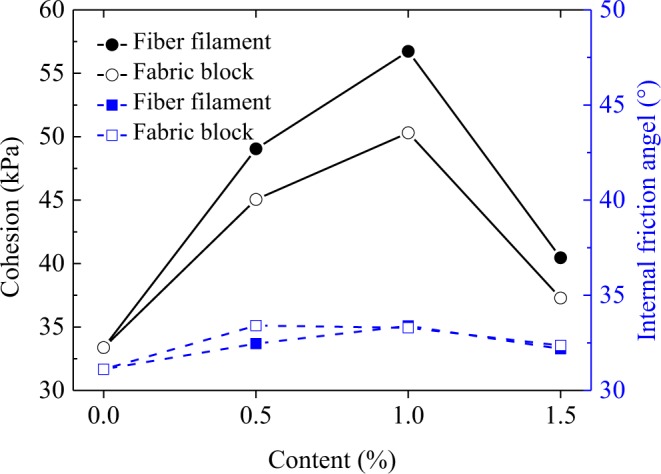
Shear strength parameter variations of two types of reinforced clay with fiber filament and fabric block contents.

The failure envelopes of clay, waste polyester fiber filament reinforced clay, and waste polyester fabric block reinforced clay are shown in Fig. 7(a,b), The deviatoric and average stresses at failure used to plot the envelopes of the shear strengths were obtained from Eqs. (1) and (2), respectively:

1 $$q={\sigma {\prime} }_{1}-{\sigma {\prime} }_{3}$$

2 $$p{\prime} =\frac{{\sigma {\prime} }_{1}+2{\sigma {\prime} }_{3}}{3}$$

where *q* is the deviatoric stress at failure, *P*′ is the mean stress, ${\sigma {\prime} }_{1}$ is the effective maximum principal stress, and ${\sigma {\prime} }_{3}$ is the effective minimum principal stress. According to^29^, the shear strengths parameters were determined by linear fitting. Figure 8 revealed that as the content of fiber filament and fabric block increased from 0.0% to 1.5%, the cohesions of both reinforced clays first increased then decreased. When the content was 1.0%, the cohesion of both reinforced clays reached their maximum values (56.73 kPa and 50.31 kPa respectively); compared with the cohesion of clay (33.38 kPa), the cohesion of the above two reinforced clays increased by 70.0% and 50.7%, respectively. This confirmed that the optimal content of fiber filament and fabric block was 1.0%. In addition, Fig. 8 shows that at the same content value, the cohesion of fiber filament reinforced clay was larger than that of fabric block reinforced clay. Comprehensively considering both the stiffness and energy absorption capacity variations of the two types of reinforced clays, the fiber filament had a better clay reinforcement effect than the fabric block.

Previous studies indicated that the mechanical behavior of the strengthened clay is closely related to the contact area between the material and the soil^1,2,5^. When the mass of the waste polyester fiber filaments and waste polyester fabric blocks are the same, that is, the content is the same, it is obvious that the contact area between the fiber filaments and the soil is larger than that of the fabric blocks. Therefore, waste polyester fiber filaments reinforced clay have better mechanical properties.

### Mathematical model of the nonlinear stress-strain relationship of reinforced clay

Triaxial test results show that the stress-strain relationship curves of clay, waste polyester fiber filament reinforced clay, and waste polyester fiber reinforced clay had obvious nonlinear characteristics. By referring to the study of^30^, a hyperbolic model was used to quantitatively describe the nonlinear stress-strain relationship. Duncan *et al*. 1970 noted that the actual stress-strain relationship curve did not exactly match the assumed hyperbolic model. In the initial loading and near-destruction stage, the test data might not fit the linear relationship when converting the ${\sigma }_{1}-{\sigma }_{3} \sim {\varepsilon }_{1}$ hyperbolic relationship into the ${\varepsilon }_{1}/({\sigma }_{1}-{\sigma }_{3}) \sim {\varepsilon }_{1}$ linear relationship^31^. Therefore, the test data in the strain range of 1.0% to 15.0% were selected for hyperbolic fitting. The hyperbola is expressed as follows:

3 $${\sigma }_{1}-{\sigma }_{3}=\frac{{\varepsilon }_{1}}{{\rm{a}}+{\rm{b}}{\varepsilon }_{1}}$$

where ${\sigma }_{1}-{\sigma }_{3}$ is the deviatoric stress, *ε*_1_ is the corresponding axial strain, and a and b are test parameters determined by converting Eq. (3) into Eq. (4) as shown below:

4 $$\frac{{\varepsilon }_{1}}{{\sigma }_{1}-{\sigma }_{3}}={\rm{a}}+{\rm{b}}{\varepsilon }_{1}$$

Equation (4) shows that the test parameters a and b can be obtained by linear fitting when knowing the test results, where a is the intercept and b is the slope of the fitted line.

**Figure 9:**
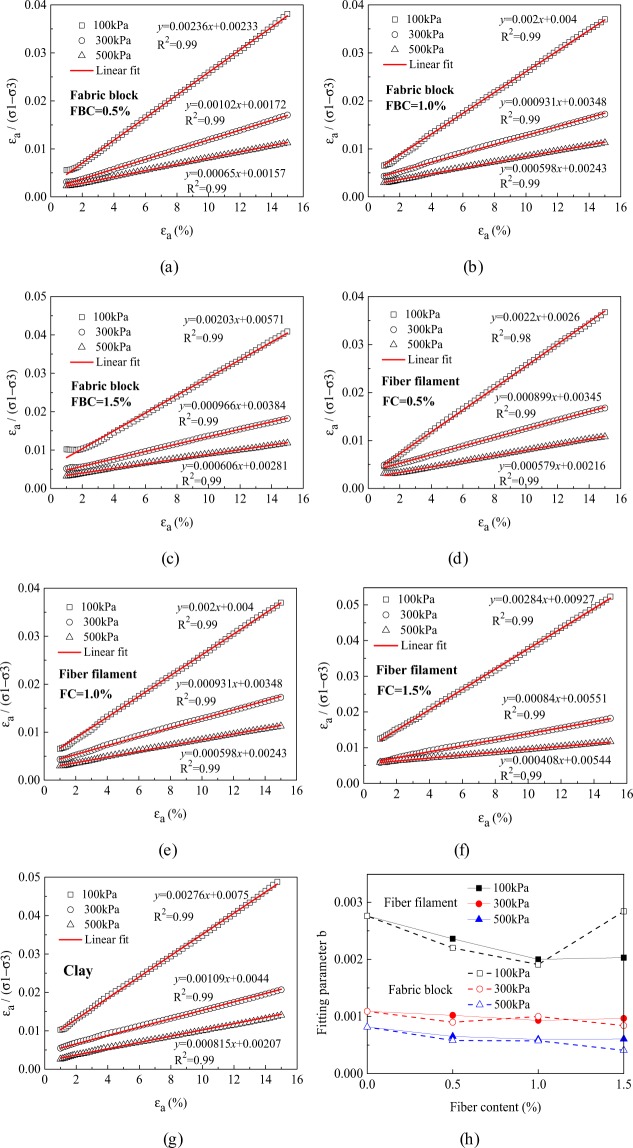
Hyperbolic fitting results and variations of fitting parameter b for the stress-strain relationship curves of clay and two types of reinforced clays. (**a**) Clay (**b**) 0.5% of fiber filaments (**c**) 1.0% of fiber filaments (**d**) 1.5% of fiber filaments (**e**) 0.5% of fabric blocks (**f**) 1.0% of fabric blocks (**g**) 1.5% of fabric blocks (**h**) variation of fitting parameter b.

Figure 9a–g shows the fitting results, and Fig. 9(h) shows the variation of the test parameter b. When the confining pressure was 100 kPa, and the fiber filament content was 1.0% or the fabric block content was 0.5%, the data in the strain range of 1.0–2.0% did not fit linearly. However, when the confining pressure increased to 300 kPa or 500 kPa, or when the strain was larger than 2%, the data were a good linear fit. In addition, when using the hyperbolic model to fit the stress-strain relationship of waste polyester fiber filament and fabric block reinforced clays, the correlation coefficients were >0.99, indicating that the fitting results were valid. Based on Fig. 9(h), at a fixed fiber filament or fabric block content, the parameter b decreased with increasing confining pressure. When the confining pressure was constant, the parameter b decreased with increasing content. When both the content and the confining pressure were fixed, the overall value of parameter b for fiber filament reinforced clay was larger than that for fabric block reinforced clay. According to the stress-strain relationship curves of clay and the two types of reinforced clays, the changes of parameter b could reflect the strain hardening degree of the samples, where a smaller value of b corresponded to a more obvious strain hardening characteristic. This provided an effective method for the quantitative evaluation of the strain hardening of clay, waste polyester fiber filament reinforced clay, and waste polyester fabric block reinforced clay.

## Conclusions

The influence of reinforcement methods and contents of waste polyester fiber or waste polyester fabric block on the deformation resistance and shear characteristics of the reinforced clay were studied by carrying out 21 groups of triaxial tests. The conclusions of the study can be summarized as follows:

1. At a constant confining pressure, the stiffness of reinforced clay decreased with increasing contents of fiber filaments or fabric blocks. When the contents of fiber filaments or fabric blocks were fixed, both stiffness and energy absorption capacity of the reinforced clays increased with increasing confining pressure.
2. The optimal content of both waste polyester fiber and waste polyester fiber fabric for the best reinforcement effects was 1.0%, and the fiber filaments showed better reinforcement effects than the fabric blocks. The cohesion of fiber filament and fabric block reinforced clays were greater than the cohesion of clay, as was the energy absorption capacity for the above two reinforced clays compared to that of clay.
3. A hyperbolic model quantitatively described the strain-hardening type of stress-strain curves of clay, waste polyester fiber reinforced clay, and waste polyester fiber fabric reinforced clay. In addition, the mathematical model of the stress-strain relationship of clay and the two types of reinforced clays under strain hardening conditions was given, with ideal results for the clays examined in this study.

The significance of this study is to serve as an important reference for the application of waste fiber and waste fiber fabric blocks in the harmless and green reinforcement of clay, and to provide an effective method for the proper disposal of waste textile fabrics. Future studies of this topic should focus on the influences of clay moisture content, dry density, particle size, and the type and size of waste fiber fabric block. In addition, whether or not the mathematical model in this study would be suitable for other types of reinforced clays still requires further experimental verification.

## Data availability

The data used to support the findings of this study are available from the corresponding author upon request.

## References

[1] Tang C, Shi B, Gao W (2007). Strength and mechanical behavior of short polypropylene fiber reinforced and cement stabilized clayey soil. Geotextiles and Geomembranes.

[2] Tang CS, Gu K (2011). Strength behavior of polypropylene fiber reinforced cement stabilized soft soil. China Civ. Eng. J..

[3] Mitchell, J. M., Jardine, F. M. *A guide to ground treatment*, *Construction Industry Research & Information Association (CIRIA)**Publications, London* (2002).

[4] Cai Y, Shi B, Ng CWW, Tang C (2006). Effect of polypropylene fiber and lime admixture on engineering properties of clayey soil. J. Eng. Geol..

[5] Chen M (2015). Laboratory evaluation on the effectiveness of polypropylene fibers on the strength of fiber-reinforced and cement-stabilized Shanghai soft clay. Geotextiles & Geomembranes.

[6] Tang, A. *et al*. Desiccation cracking behavior of polypropylene fiber–reinforced clayey soil. *Canadian Geotech. J*. **49**(9), 1088–1101 (2012).

[7] Van Impe, W. F. *Soil improvement techniques and their evolution*, A.A. Balkema, Rotterdam, Netherlands (1989).

[8] Mirzababaei M, Miraftab M, Mohamed M, McMahon P (2013). Unconfined Compression Strength of Reinforced Clays with Carpet Waste Fibers. J. Geotechn. Geoenv. Eng..

[9] Yetimoglu T, Salbas O (2003). A study on shear strength of sands reinforced with randomly distributed discrete fibers. J. Geotextiles Geomembranes.

[10] Liu B (2013). Advances in engineering properties of fiber reinforced soil. J. Eng. Geol..

[11] Sharma V, Vinayak HK, Marwaha BM (2015). Enhancing compressive strength of soil using natural fibers. Const. Build. Mater..

[12] Gao L, Hu GH, Chen YH, Hu YJ, Gong YH (2017). Triaxial tests clay reinforced by basalt fiber. Chinese J. Geotech. Eng..

[13] Cao LT, Ren HL, Zuo JH, Du QL (2014). Mechanical properties of textile fiber modified clay. J. Build. Mater..

[14] Estabragh AR, Namdar P, Jawadi AA (2012). Behavior of cement-stabilized clay reinforced with nylon fiber. Geosynthetics Int..

[15] Consoli NC, Montardo JP, Priette PDM, Pasa GS (2002). Engineering behaviour of a sand reinforced with plastic waste. J. Geotech. Geoenv. Eng..

[16] Foose GJ, Benson CH, Bosscher PJ (1996). Sand reinforced with shredded waste tires. J. Geotech. Eng. ASCE.

[17] Hataf N, Rahimi MM (2006). Experimental investigation of bearing capacity of sand reinforced with randomly distributed tire shreds. Constr. Build. Mater..

[18] Maryam S, Mohammadali R, Sayyed MA, Sayyed MH (2017). Optimization of carpet waste fibers and steel slag particles to reinforce expansive soil using response surface methodology. Applied Clay Science,.

[19] Thyagaraj T, Soujanya D (2017). Polypropylene fiber reinforced bentonite for waste containment barriers. Applied Clay Science,.

[20] Liu YY, Zhang YH, Guo YX, Chu PK, Tu SC (2017). Porous materials composed of flue gas Desulfurization Gypsum and Textile Fiber Wastes. Waste and Biomass Valorization,.

[21] ASTM D 2487. Standard Practice for Classification of Soils for Engineering Purposes, ASTM International, West Conshohocken, PA, USA. (2000).

[22] ASTM D 4318-93, Standard Test Method for Liquid Limit, Plastic Limit and Plasticity Index of Soils. ASTM International, West Conshohocken, *PA, USA* (1994).

[23] ASTM D 422-63, Standard Test Method for Particle Size Analysis of Soils. ASTM International, West Conshohocken, *PA, USA* (1994).

[24] ASTM D1577-07, Standard Test Methods for Linear Density of Textile Fibers. ASTM International, West Conshohocken, *PA, USA* (2012).

[25] ASTM D3822/D3822M-14, *Standard Test Method for Tensile Properties of Single Textile* (2014).

[26] Hamidi A, Hooresfand M (2013). Effect of fiber reinforcement on triaxial shear behavior of cement treated sand. Geotextiles and Geomembranes.

[27] Ladd RS (1978). Preparing test specimens using under compaction. Geotech. Test. J. ASTM.

[28] IS: 2720-7. Determination of water content-dry density relation using. Indian Standards; (1985).

[29] Consoli NC, Prietto P, Ulbrich LA (1998). Influence of fiber and cement addition on behavior of sandy soil. J. Geotech. Geoenv. Eng..

[30] Kondner RL (1963). Hyperbolic Stress-Strain Response: Cohesive Soils. J. Soil Mech. Found. Div. ASCE.

[31] Duncan JM, Chang CY (1970). Nonlinear analysis of stress and strain in soils. J. Soil Mech. Found. Div. ASCE.

